# Refractory Trigeminal Neuralgia: The Role of MRI in Diagnosing Neurovascular Compression

**DOI:** 10.31083/RN42818

**Published:** 2025-10-31

**Authors:** Dalmo José Gonçalves Netto Borges, Marcelo de Queiroz Pereira da Silva, Bruno Fernandes Barros Brehme de Abreu, Thaís Nogueira Dantas Gastaldi, Márcio Luís Duarte

**Affiliations:** ^1^Department of Internal Medicine, Faculdade de Ciências Médicas de Santos, 11045-101 Santos (SP), Brazil; ^2^Department of Radiology, WEBIMAGEM Telerradiologia, 01139-020 São Paulo (SP), Brazil; ^3^Department of Radiology, Diagnósticos da América S/A – DASA, 05425-070 São Paulo (SP), Brazil; ^4^Department of Radiology, Universidade de Ribeirão Preto – Campus Guarujá, 11440-003 Guarujá (SP), Brazil

**Keywords:** magnetic resonance imaging, trigeminal neuralgia, nerve injuries, resonancia magnética, neuralgia del trigémino, lesiones nerviosas

A 61-year-old woman presented with severe, electric shock-like pain on the left side of the face, predominantly in the maxillary region, lasting a few seconds but of excruciating intensity and limiting daily activities. She had a 10-year history of trigeminal neuralgia and had undergone microvascular decompression 15 months earlier, with only partial and transient improvement. At the time of presentation, she was using carbamazepine and gabapentin with limited efficacy. Her past medical history included type 2 diabetes mellitus, controlled with diet, and allergy to benzathine benzylpenicillin. Neurological examination revealed no focal deficits. Magnetic resonance imaging (MRI) demonstrated that the left anterior inferior cerebellar artery crossed the medial border and superior aspect of the trigeminal nerve root, with mild signal changes suggesting neurovascular conflict at the level of the Gasserian ganglion. In addition, the superior cerebellar artery (SCA) was also seen in close relation to the nerve, a finding frequently described in the literature, although in this case the conflict was primarily associated with the anterior inferior cerebellar artery (AICA) (Fig. 1). The patient was referred for repeat neurosurgical evaluation.

**Fig. 1.**
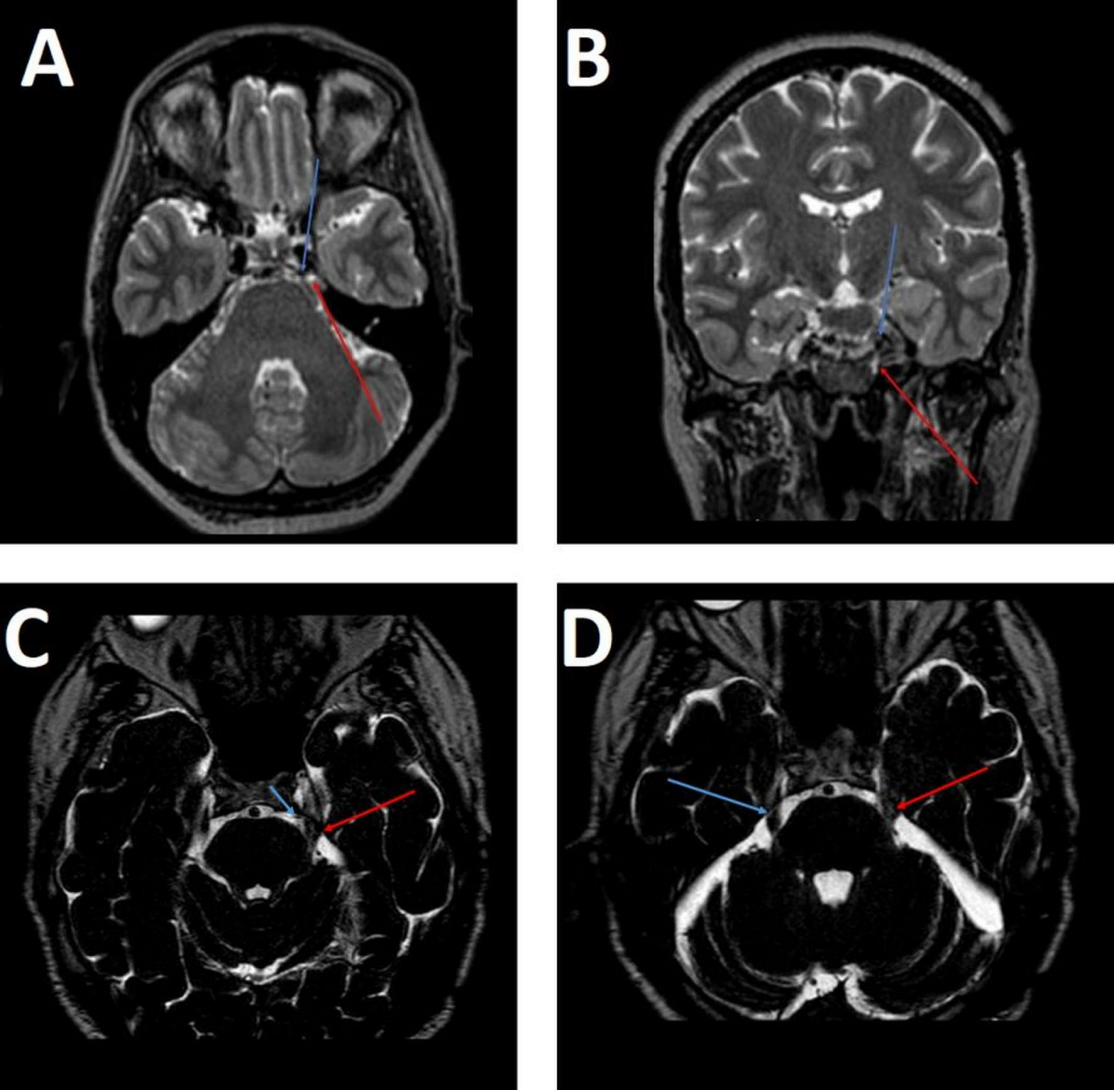
**High-resolution T2-weighted magnetic resonance imaging (MRI)**. (A) Axial and (B) coronal volumetric sequences demonstrate neurovascular conflict between the left superior cerebellar artery (blue arrow) and the left trigeminal nerve (red arrow), with subtle signal alteration at the root entry zone, consistent with neuropathy. (C) Axial T2 3D sequence shows the left anterior inferior cerebellar artery (blue arrow) crossing the ipsilateral trigeminal nerve (red arrow), which appears diffusely thickened and with altered signal. (D) Axial T2 3D sequence demonstrates thickening and signal alteration of the left trigeminal nerve (red arrow) compared with the contralateral side (blue arrow), corresponding to Sindou grade II (morphological change without severe displacement). Note: Time-of-Flight (TOF) angiography and post-contrast T1-weighted sequences were not acquired in this case.

Trigeminal neuralgia remains one of the most severe pain syndromes and is often associated with neurovascular compression, most frequently by the superior cerebellar artery [1, 2, 3]. However, it is important to distinguish between mere vascular contact and true neurovascular conflict, since not every contact requires surgical intervention. The presence of nerve displacement, distortion, or focal hyperintensity on T2-weighted imaging correlates more strongly with clinically relevant compression and surgical benefit [4].

Several classifications have been proposed to grade neurovascular conflicts, with the Sindou system being one of the most widely adopted. This classification stratifies conflicts into contact without displacement, displacement without distortion, and severe distortion or indentation of the nerve, which is most predictive of symptomatic disease and surgical success [5]. Incorporating these criteria into radiological reports may help prevent unnecessary microvascular decompressions in patients with incidental vascular contact.

Magnetic resonance imaging, particularly with high-resolution 3D T2-weighted sequences combined with angiographic techniques, remains the cornerstone for diagnosis. Emerging techniques, such as diffusion tensor imaging (DTI), have shown promise in detecting microstructural abnormalities of the trigeminal nerve, including decreased fractional anisotropy, which correlates with demyelination and symptom severity [6]. Although not yet standard in clinical practice, DTI may represent a valuable adjunct for patient selection and prognostic assessment. In the present case, only volumetric T2-weighted sequences were available. Time-of-flight angiography and post-contrast T1-weighted images, although generally recommended, were not performed.

In the present case, MRI not only demonstrated vascular contact but also subtle signal alteration at the trigeminal root entry zone, supporting the diagnosis of true neurovascular conflict. This highlights the critical role of MRI in guiding surgical decision-making, especially in recurrent or refractory cases. Additional quiz questions are available in the **Supplementary Material**.

## Availability of Data and Materials

The authors state that they have followed the protocols of their Center and Local regulations on the publication of patient data. The datasets are available from the corresponding author on reasonable request.

## Supplementary Material

Supplementary material associated with this article can be found, in the online version, at https://doi.org/10.31083/RN42818.

## References

[1] Miki K, Natori Y, Mori M, Kai Y, Yamada T, Noguchi N (2019). Trigeminal Neuralgia Caused by a Persistent Primitive Trigeminal Artery Variant and Superior Cerebellar Artery. *NMC Case Report Journal*.

[2] Hardy DG, Rhoton AL (1978). Microsurgical relationships of the superior cerebellar artery and the trigeminal nerve. *Journal of Neurosurgery*.

[3] Malicki M, Szmyd BM, Bobeff EJ, Karuga FF, Piotrowski MM, Kościołek D (2023). The Superior Cerebellar Artery: Variability and Clinical Significance. *Biomedicines*.

[4] Satoh T, Onoda K, Date I (2007). Fusion imaging of three-dimensional magnetic resonance cisternograms and angiograms for the assessment of microvascular decompression in patients with hemifacial spasms. *Journal of Neurosurgery*.

[5] Sindou M, Leston J, Decullier E, Chapuis F (2007). Microvascular decompression for primary trigeminal neuralgia: long-term effectiveness and prognostic factors in a series of 362 consecutive patients with clear-cut neurovascular conflicts who underwent pure decompression. *Journal of Neurosurgery*.

[6] DeSouza DD, Hodaie M, Davis KD (2014). Abnormal trigeminal nerve microstructure and brain white matter in idiopathic trigeminal neuralgia. *Pain*.

