# Supplementary material for: Refractory Trigeminal Neuralgia: The Role of MRI in Diagnosing Neurovascular Compression
Source: Rev Neurol. 2025 Oct 31;80(9):42818. doi: 10.31083/RN42818 (PMC12593730; doi:10.31083/RN42818)
Supplement: Supplementary file 1 [file 1576-6578-80-9-42818-s1.pdf]

Questions:

1. What is the main etiological hypothesis for trigeminal neuralgia described in the text?

A) Chronic viral infection
B) Complications from diabetes mellitus
C) Neurovascular compression by blood vessels
D) Previous cranial trauma
E) Autoimmune demyelinating disease

Correct answer: C) Neurovascular compression by blood vessels

Explanation:
The text emphasizes that trigeminal neuralgia is most commonly caused by compression of the trigeminal nerve by blood vessels, especially the superior cerebellar artery.

2. Which artery is most commonly implicated in compressing the trigeminal nerve?

A) Middle cerebral artery
B) Internal carotid artery
C) Vertebral artery
D) Superior cerebellar artery
E) Basilar artery

Correct answer: D) Superior cerebellar artery

Explanation:
The superior cerebellar artery (SCA) is identified in the text as the vessel most often associated with trigeminal nerve compression due to its tortuous and elongated path.

3. What is the primary imaging modality used to diagnose neurovascular compression in trigeminal neuralgia?

A) Computed tomography (CT)
B) Doppler ultrasound
C) Skull X-ray
D) Electroencephalogram (EEG)
E) Magnetic resonance imaging (MRI)

Correct answer: E) Magnetic resonance imaging (MRI)

Explanation:
The text states that MRI is essential for diagnosing trigeminal neuralgia, as it can identify nerve compression and rule out other conditions such as tumors or aneurysms.

4. What treatment is recommended for refractory cases of trigeminal neuralgia?

A) Acupuncture
B) Physical therapy
C) Microvascular decompression (MVD)
D) Local anesthetic block
E) Increasing the dose of gabapentin

Correct answer: C) Microvascular decompression (MVD)

Explanation:
For patients who do not respond to medications like carbamazepine, microvascular decompression (MVD) is the recommended treatment, as it aims to relieve the pressure on the nerve.

5. Which combination of MRI sequences is considered most effective for assessing neurovascular conflict in trigeminal neuralgia?

A) Plain T1 and FLAIR
B) Axial T2, DWI, and brain perfusion
C) High-resolution 3D T2, 3D TOF angiography, and gadolinium-enhanced T1
D) Coronal T2 and iodinated contrast angiography
E) Non-contrast T1, SWI, and MR spectroscopy

Correct answer: C) High-resolution 3D T2, 3D TOF angiography, and gadolinium-enhanced T1

Explanation:
The text recommends a combination of high-resolution 3D T2-weighted imaging, 3D time-of-flight (TOF) angiography, and gadolinium-enhanced T1 sequences as the most effective for detecting neurovascular conflict.
